# Supplementary material for: Single-cell RNA-sequencing of peripheral blood mononuclear cells reveals widespread, context-specific gene expression regulation upon pathogenic exposure
Source: Nat Commun. 2022 Jun 7;13:3267. doi: 10.1038/s41467-022-30893-5 (PMC9174272; doi:10.1038/s41467-022-30893-5)
Supplement: Supplementary file 1 — Supplementary Information [file 41467_2022_30893_MOESM1_ESM.pdf]

Supplementary Information

Single-cell RNA-sequencing of peripheral blood mononuclear cells reveals widespread, context-specific gene expression regulation upon pathogenic exposure.

Roy Oelen[1,2#], Dylan H. de Vries[1,2#], Harm Brugge[1,2#], M. Grace Gordon[3-6], Martijn Vochteloo[1], single-cell eQTLGen consortium[$], BIOS Consortium[$], Chun J. Ye[4,6-10], Harm-Jan Westra[1], Lude Franke[1,2*`], Monique G.P. van der Wijst[1,2*`]

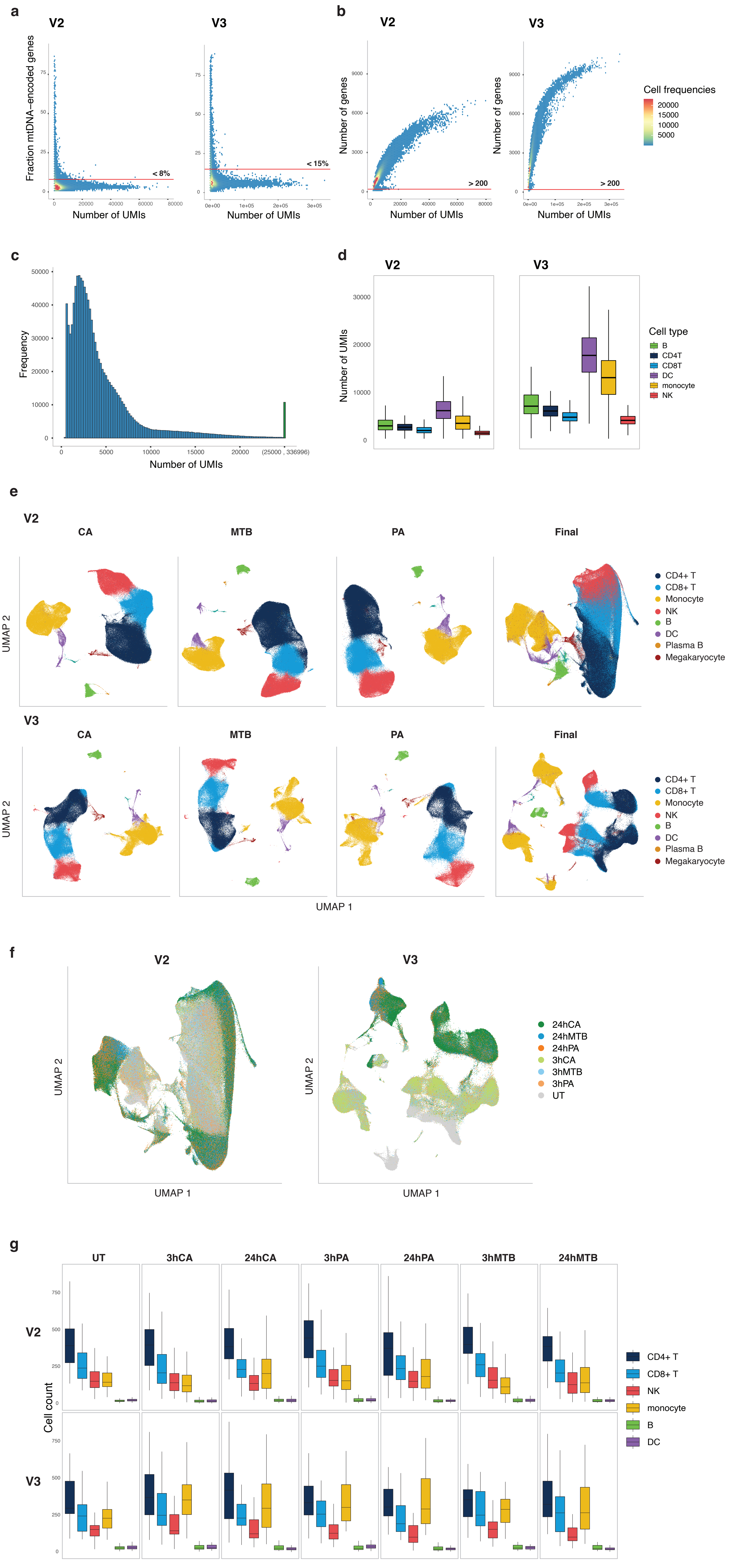

a
V2
V3
Fraction mtDNA-encoded genes
Number of UMIs
< 8%
< 15%
b
V2
V3
Number of genes
Number of UMIs
> 200
Cell frequencies
20000
15000
10000
5000
c
Frequency
Number of UMIs
(25000 , 336996)
d
V2
V3
Number of UMIs
Cell type
B
CD4T
CD8T
DC
monocyte
NK
e
V2
CA
MTB
PA
Final
V3
UMAP 2
UMAP 1
CD4+ T
CD8+ T
Monocyte
NK
B
DC
Plasma B
Megakaryocyte
f
V2
V3
UMAP 2
UMAP 1
24hCA
24hMTB
24hPA
3hCA
3hMTB
3hPA
UT
g
UT
3hCA
24hCA
3hPA
24hPA
3hMTB
24hMTB
V2
V3
Cell count
CD4+ T
CD8+ T
NK
monocyte
B
DC

Supplementary Figure 1. Dataset characteristics
a. *The proportion of mitochondrial genes per cell (y-axis) against the number of unique molecular identifiers (UMIs) per cell (x-axis), split per scRNA-seq library chemistry (v2 or v3). Cell density is indicated by color in each graph, going from blue to red for low to high cell numbers, respectively. The red line indicates the QC threshold used for removing cells.* ***b.*** *Number of expressed genes versus number of UMIs per cell. Cell density is indicated by color in each graph, going from blue to red for low to high cell numbers, respectively. The red line indicates the QC threshold used for removing cells with a low number of expressed genes.* ***c.*** *Distribution of the number of UMIs observed per cell (combining v2 and v3 chemistry). Note that the rightest bin (green) is larger than the other ones.* ***d.*** *Boxplot (showing median, 25th and 75th percentile, and 1.5 x the interquartile range) of the observed number of UMIs per cell, split by cell type and library chemistry.* ***e.*** *The UMAP plots per stimulation and library chemistry. Each dot represents a single-cell and the color indicates the assigned cell type.* ***f.*** *The integrated UMAP per chemistry where all cells are combined.* ***g.*** *Boxplots (showing median, 25th and 75th percentile, and 1.5 x the interquartile range) representing the cell type proportions per individual, split by stimulation-timepoint combination and chemistry. Colors represent the cell types. The number of individuals and cells included in each analysis can be found in the Source Data file.*

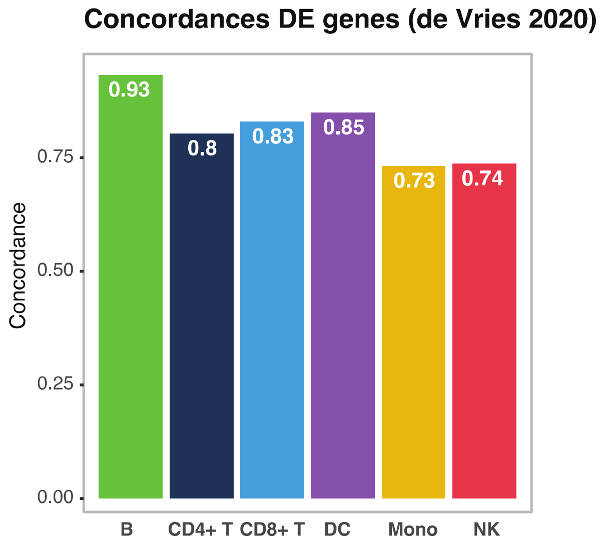

Supplementary Figure 2. Concordance differential expression results with literature
Bar plot showing the concordance of the identified differentially expressed (DE) genes in this study (as shown in ***Supplementary Data 5****) with those identified in de Vries et al. 2020. Each bar represents a different cell type. The number of individuals and cells included in each analysis can be found in the Source Data file.*

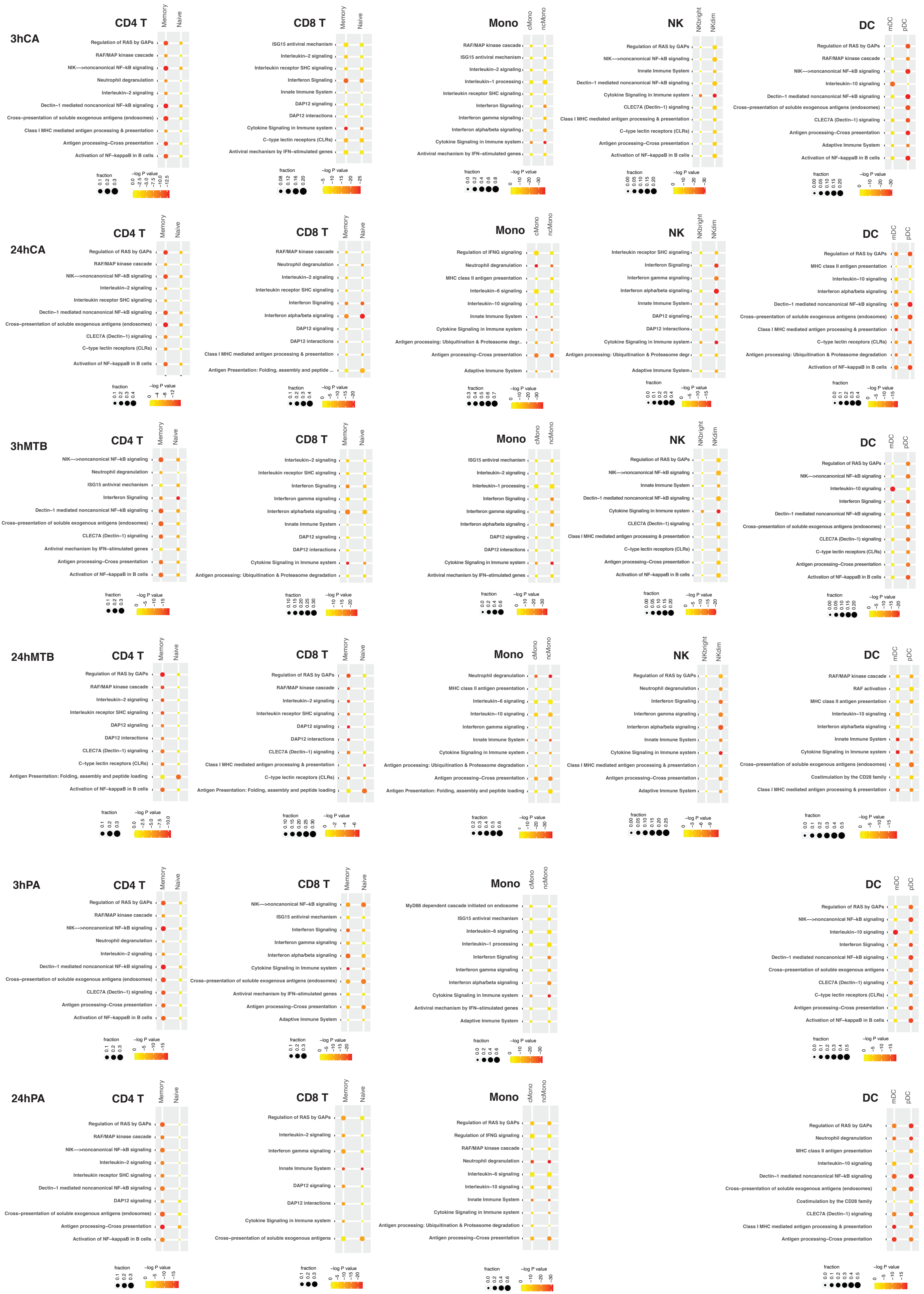

3hCA
CD4 T
Memory
Naive
Regulation of RAS by GAPs
RAF/MAP kinase cascade
NIK-->noncanonical NF-kB signaling
Neutrophil degranulation
Interleukin-2 signaling
Dectin-1 mediated noncanonical NF-kB signaling
Cross-presentation of soluble exogenous antigens (endosomes)
Class I MHC mediated antigen processing & presentation
Antigen processing-Cross presentation
Activation of NF-kappaB in B cells
fraction
-log P value
CD8 T
ISG15 antiviral mechanism
Interleukin-2 signaling
Interleukin receptor SHC signaling
Interferon Signaling
Innate Immune System
DAP12 signaling
DAP12 interactions
Cytokine Signaling in Immune system
C-type lectin receptors (CLRs)
Antiviral mechanism by IFN-stimulated genes
Mono
cMono
ncMono
RAF/MAP kinase cascade
ISG15 antiviral mechanism
Interleukin-2 signaling
Interleukin-1 processing
Interleukin receptor SHC signaling
Interferon Signaling
Interferon gamma signaling
Interferon alpha/beta signaling
Cytokine Signaling in Immune system
Antiviral mechanism by IFN-stimulated genes
NK
NKbright
NKdim
Regulation of RAS by GAPs
NIK-->noncanonical NF-kB signaling
Innate Immune System
Dectin-1 mediated noncanonical NF-kB signaling
Cytokine Signaling in Immune system
CLEC7A (Dectin-1) signaling
Class I MHC mediated antigen processing & presentation
C-type lectin receptors (CLRs)
Antigen processing-Cross presentation
Activation of NF-kappaB in B cells
DC
mDC
pDC
Regulation of RAS by GAPs
RAF/MAP kinase cascade
NIK-->noncanonical NF-kB signaling
Interleukin-10 signaling
Dectin-1 mediated noncanonical NF-kB signaling
Cross-presentation of soluble exogenous antigens (endosomes)
CLEC7A (Dectin-1) signaling
Antigen processing-Cross presentation
Adaptive Immune System
Activation of NF-kappaB in B cells
24hCA
CD4 T
Regulation of RAS by GAPs
RAF/MAP kinase cascade
NIK-->noncanonical NF-kB signaling
Interleukin-2 signaling
Interleukin receptor SHC signaling
Dectin-1 mediated noncanonical NF-kB signaling
Cross-presentation of soluble exogenous antigens (endosomes)
CLEC7A (Dectin-1) signaling
C-type lectin receptors (CLRs)
Activation of NF-kappaB in B cells
CD8 T
RAF/MAP kinase cascade
Neutrophil degranulation
Interleukin-2 signaling
Interleukin receptor SHC signaling
Interferon Signaling
Interferon alpha/beta signaling
DAP12 signaling
DAP12 interactions
Class I MHC mediated antigen processing & presentation
Antigen Presentation: Folding, assembly and peptide ...
Mono
Regulation of IFNG signaling
Neutrophil degranulation
MHC class II antigen presentation
Interleukin-6 signaling
Interleukin-10 signaling
Innate Immune System
Cytokine Signaling in Immune system
Antigen processing: Ubiquitination & Proteasome degr..
Antigen processing-Cross presentation
Adaptive Immune System
NK
Interleukin receptor SHC signaling
Interferon Signaling
Interferon gamma signaling
Interferon alpha/beta signaling
Innate Immune System
DAP12 signaling
DAP12 interactions
Cytokine Signaling in Immune system
Antigen processing: Ubiquitination & Proteasome degr
Adaptive Immune System
DC
Regulation of RAS by GAPs
MHC class II antigen presentation
Interleukin-10 signaling
Interferon alpha/beta signaling
Dectin-1 mediated noncanonical NF-kB signaling
Cross-presentation of soluble exogenous antigens (endosomes)
Class I MHC mediated antigen processing & presentation
C-type lectin receptors (CLRs)
Antigen processing: Ubiquitination & Proteasome degradation
Activation of NF-kappaB in B cells
3hMTB
CD4 T
NIK-->noncanonical NF-kB signaling
Neutrophil degranulation
ISG15 antiviral mechanism
Interferon Signaling
Dectin-1 mediated noncanonical NF-kB signaling
Cross-presentation of soluble exogenous antigens (endosomes)
CLEC7A (Dectin-1) signaling
Antiviral mechanism by IFN-stimulated genes
Antigen processing-Cross presentation
Activation of NF-kappaB in B cells
CD8 T
Interleukin-2 signaling
Interleukin receptor SHC signaling
Interferon Signaling
Interferon gamma signaling
Interferon alpha/beta signaling
Innate Immune System
DAP12 signaling
DAP12 interactions
Cytokine Signaling in Immune system
Antigen processing: Ubiquitination & Proteasome degradation
Mono
ISG15 antiviral mechanism
Interleukin-2 signaling
Interleukin-1 processing
Interferon Signaling
Interferon gamma signaling
Interferon alpha/beta signaling
DAP12 signaling
DAP12 interactions
Cytokine Signaling in Immune system
Antiviral mechanism by IFN-stimulated genes
NK
Regulation of RAS by GAPs
NIK-->noncanonical NF-kB signaling
Innate Immune System
Dectin-1 mediated noncanonical NF-kB signaling
Cytokine Signaling in Immune system
CLEC7A (Dectin-1) signaling
Class I MHC mediated antigen processing & presentation
C-type lectin receptors (CLRs)
Antigen processing-Cross presentation
Activation of NF-kappaB in B cells
DC
Regulation of RAS by GAPs
NIK-->noncanonical NF-kB signaling
Interleukin-10 signaling
Interferon Signaling
Dectin-1 mediated noncanonical NF-kB signaling
Cross-presentation of soluble exogenous antigens (endosomes)
CLEC7A (Dectin-1) signaling
C-type lectin receptors (CLRs)
Antigen processing-Cross presentation
Activation of NF-kappaB in B cells
24hMTB
CD4 T
Regulation of RAS by GAPs
RAF/MAP kinase cascade
Interleukin-2 signaling
Interleukin receptor SHC signaling
DAP12 signaling
DAP12 interactions
CLEC7A (Dectin-1) signaling
C-type lectin receptors (CLRs)
Antigen Presentation: Folding, assembly and peptide loading
Activation of NF-kappaB in B cells
CD8 T
Regulation of RAS by GAPs
RAF/MAP kinase cascade
Interleukin-2 signaling
Interleukin receptor SHC signaling
DAP12 signaling
DAP12 interactions
CLEC7A (Dectin-1) signaling
Class I MHC mediated antigen processing & presentation
C-type lectin receptors (CLRs)
Antigen Presentation: Folding, assembly and peptide loading
Mono
Neutrophil degranulation
MHC class II antigen presentation
Interleukin-6 signaling
Interleukin-10 signaling
Interferon gamma signaling
Innate Immune System
Cytokine Signaling in Immune system
Antigen processing: Ubiquitination & Proteasome degradation
Antigen processing-Cross presentation
Antigen Presentation: Folding, assembly and peptide loading
NK
Regulation of RAS by GAPs
Neutrophil degranulation
Interferon Signaling
Interferon gamma signaling
Interferon alpha/beta signaling
Innate Immune System
Cytokine Signaling in Immune system
Class I MHC mediated antigen processing & presentation
Antigen processing-Cross presentation
Adaptive Immune System
DC
RAF/MAP kinase cascade
RAF activation
MHC class II antigen presentation
Interleukin-10 signaling
Interferon alpha/beta signaling
Innate Immune System
Cytokine Signaling in Immune system
Cross-presentation of soluble exogenous antigens (endosomes)
Costimulation by the CD28 family
Class I MHC mediated antigen processing & presentation
3hPA
CD4 T
Regulation of RAS by GAPs
RAF/MAP kinase cascade
NIK-->noncanonical NF-kB signaling
Neutrophil degranulation
Interleukin-2 signaling
Dectin-1 mediated noncanonical NF-kB signaling
Cross-presentation of soluble exogenous antigens (endosomes)
CLEC7A (Dectin-1) signaling
Antigen processing-Cross presentation
Activation of NF-kappaB in B cells
CD8 T
NIK-->noncanonical NF-kB signaling
ISG15 antiviral mechanism
Interferon Signaling
Interferon gamma signaling
Interferon alpha/beta signaling
Cytokine Signaling in Immune system
Cross-presentation of soluble exogenous antigens (endosomes)
Antiviral mechanism by IFN-stimulated genes
Antigen processing-Cross presentation
Adaptive Immune System
Mono
MyD88 dependent cascade initiated on endosome
ISG15 antiviral mechanism
Interleukin-6 signaling
Interleukin-1 processing
Interferon Signaling
Interferon gamma signaling
Interferon alpha/beta signaling
Cytokine Signaling in Immune system
Antiviral mechanism by IFN-stimulated genes
Adaptive Immune System
DC
Regulation of RAS by GAPs
NIK-->noncanonical NF-kB signaling
Interleukin-10 signaling
Interferon Signaling
Dectin-1 mediated noncanonical NF-kB signaling
Cross-presentation of soluble exogenous antigens
CLEC7A (Dectin-1) signaling
C-type lectin receptors (CLRs)
Antigen processing-Cross presentation
Activation of NF-kappaB in B cells
24hPA
CD4 T
Regulation of RAS by GAPs
RAF/MAP kinase cascade
NIK-->noncanonical NF-kB signaling
Interleukin-2 signaling
Interleukin receptor SHC signaling
Dectin-1 mediated noncanonical NF-kB signaling
DAP12 signaling
Cross-presentation of soluble exogenous antigens (endosomes)
Antigen processing-Cross presentation
Activation of NF-kappaB in B cells
CD8 T
Regulation of RAS by GAPs
Interleukin-2 signaling
Interferon gamma signaling
Innate Immune System
DAP12 signaling
DAP12 interactions
Cytokine Signaling in Immune system
Cross-presentation of soluble exogenous antigens
Mono
Regulation of RAS by GAPs
Regulation of IFNG signaling
RAF/MAP kinase cascade
Neutrophil degranulation
Interleukin-6 signaling
Interleukin-10 signaling
Innate Immune System
Cytokine Signaling in Immune system
Antigen processing: Ubiquitination & Proteasome degradation
Antigen processing-Cross presentation
DC
Regulation of RAS by GAPs
Neutrophil degranulation
MHC class II antigen presentation
Interleukin-10 signaling
Dectin-1 mediated noncanonical NF-kB signaling
Cross-presentation of soluble exogenous antigens (endosomes)
Costimulation by the CD28 family
CLEC7A (Dectin-1) signaling
Class I MHC mediated antigen processing & presentation
Antigen processing-Cross presentation

Figure S3. Most differentially enriched pathways in the subcell types
Dotplots of the 10 most enriched pathways with the largest difference in significance between both subcell types, split by subcell type and pathogen stimulation (a complete overview of the enriched pathways can be found in ***Table S6****). When subcell type classification provided too few cells or could not be confidently made, no results are shown for that pathogen stimulation. The color indicates the log10 transformed FDR p-value, the size of the dot represents the fraction of DE genes that were found in the total list of genes for the pathway. The number of individuals and cells included in each analysis can be found in the Source Data file.*

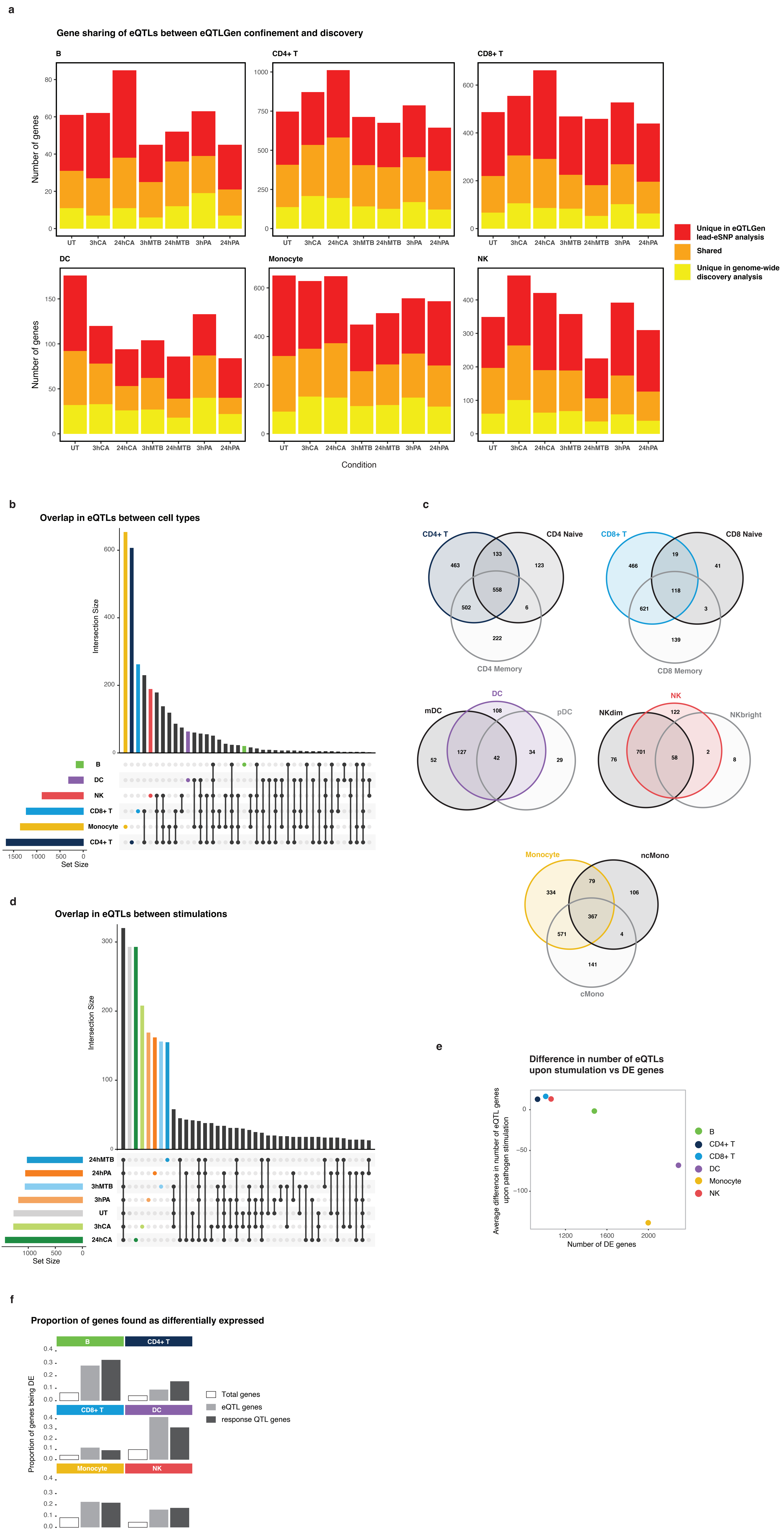

a
Gene sharing of eQTLs between eQTLGen confinement and discovery
B
CD4+ T
CD8+ T
DC
Monocyte
NK
Number of genes
UT
3hCA
24hCA
3hMTB
24hMTB
3hPA
24hPA
Condition
Unique in eQTLGen lead-eSNP analysis
Shared
Unique in genome-wide discovery analysis
b
Overlap in eQTLs between cell types
Intersection Size
Set Size
B
DC
NK
CD8+ T
Monocyte
CD4+ T
c
CD4+ T
CD4 Naive
CD4 Memory
CD8+ T
CD8 Naive
CD8 Memory
DC
mDC
pDC
NK
NKdim
NKbright
Monocyte
ncMono
cMono
d
Overlap in eQTLs between stimulations
Intersection Size
Set Size
24hMTB
24hPA
3hMTB
3hPA
UT
3hCA
24hCA
e
Difference in number of eQTLs upon stimulation vs DE genes
Average difference in number of eQTL genes upon pathogen stimulation
Number of DE genes
B
CD4+ T
CD8+ T
DC
Monocyte
NK
f
Proportion of genes found as differentially expressed
Proportion of genes being DE
B
CD4+ T
CD8+ T
DC
Monocyte
NK
Total genes
eQTL genes
response QTL genes

Supplementary Figure 4. eQTL characteristics

a. *Stacked bar plots showing the total number of unique eQTL genes per stimulation-timepoint combination within the eQTLGen lead-eSNP confined eQTL analysis (**Supplementary Data 7**) and the genome-wide cis-eQTL discovery analysis (**Supplementary Data 8**). The red color shows eQTL genes that were uniquely identified in the eQTLGen lead-eSNP confined analysis, orange is shared across both analyses and yellow shows eQTL genes that are unique to the genome-wide cis-eQTL discovery analysis.* ***b.*** *Overlap of eQTL effects between cell types. Each bar represents the number of eQTLs found for that group, which is indicated by the dots underneath the bar. Colored bars show eQTLs that are unique to one group and black bars are a combination of different groups.* ***c.*** *Venn diagrams that show the overlap in identified eQTL genes (in the eQTLGen-confined eQTL analysis) for each major cell type and their two corresponding subcell types.* ***d.*** *Overlap of eQTL effects between stimulation-timepoint combinations. Each bar represents the number of eQTLs found for that group, which is indicated by the dots underneath the bar. Colored bars show eQTLs that are unique to one group and black bars are a combination of different groups.* ***e.*** *Dot plot showing the difference in number of identified eQTLs upon stimulation (y-axis) compared to the number of DE genes (x-axis). Each dot represents a different cell type, shown by the color.* ***f.*** *Bar plots showing the proportion of genes that were identified as DE (**Supplementary Data 5**) per cell type. The three bars represent the complete set of tested genes (white), the complete set of genes with at least one eQTL (gray, **Supplementary Data 7**) and the complete set of genes with at least one response QTL (dark gray, **Supplementary Data 9**). The number of individuals and cells included in each analysis can be found in the Source Data file.*

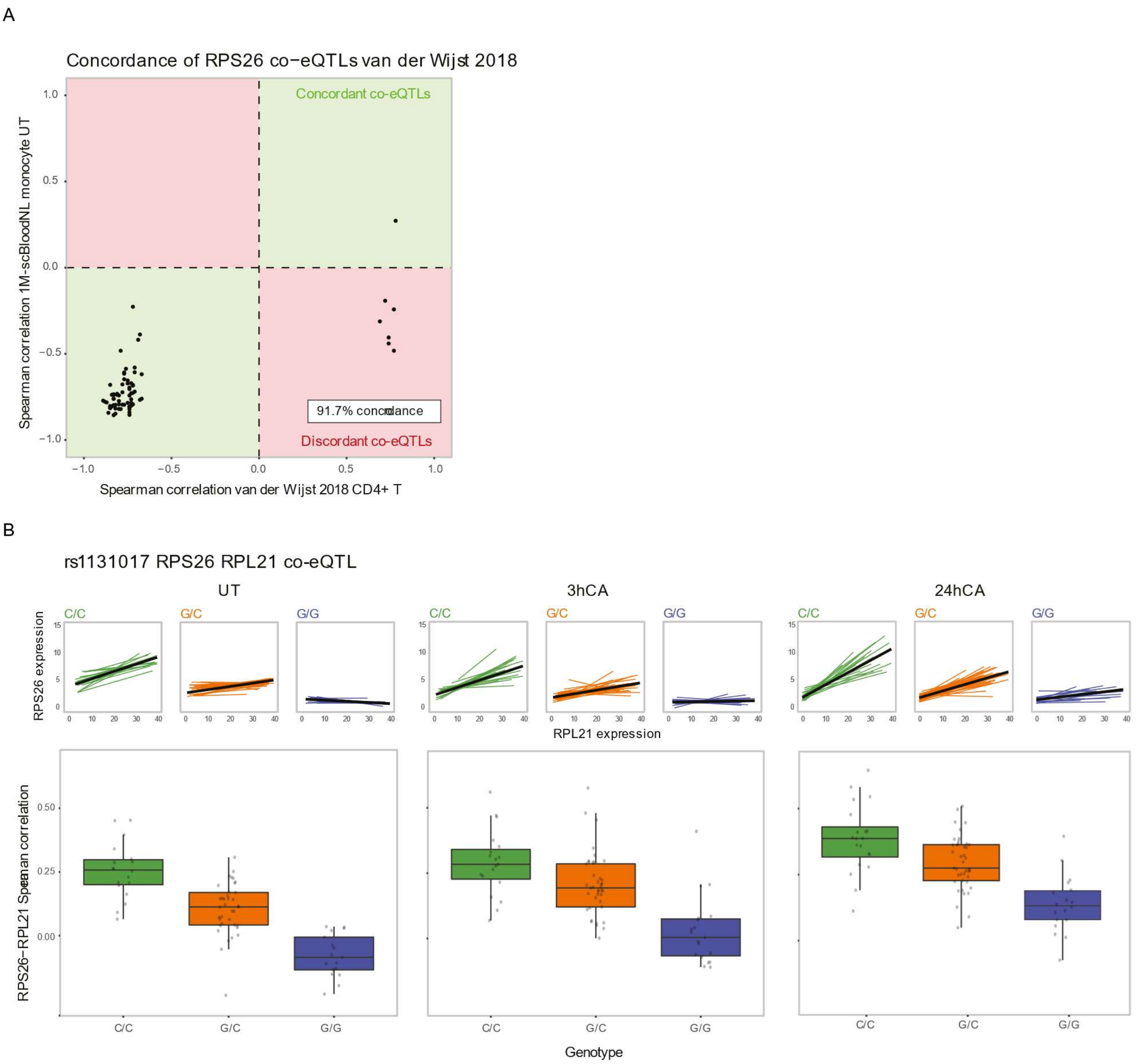

Supplementary Figure 5. RPS26 co-expression QTLs

a. *Concordance plot comparing Spearman correlations of RPS26 co-expression QTLs in CD4+ T cells from van der Wijst et al. 2018 (x-axis) compared to RPS26 co-expression QTLs in monocytes in the untreated condition of this study. Each dot represents a different co-expression QTL. Dots in green quadrants are concordant and dots in red quadrants are discordant.* ***b.*** *The co-expression QTL of RPS26 and RPL21, mediated by rs1131018, across different stimulation-timepoint combinations (meta-analysis p for the UT 1.21x10^{-31}, 3h CA 4.72x10^{-24}, 24h CA 1.28x10^{-5} conditions. Full summary statistics can be found in* ***Supplementary Data 11****). The top graphs show the individual co-expression, with each colored line representing one individual and the black line showing the regression line across all points. Boxplots (showing median, 25th and 75th percentile, and 1.5 x the interquartile range) representing the Spearman correlations per individual, split by genotype group. Each dot represents the Spearman correlation between RPS26 and RPL21 for one individual within that genotype group. Colors represent the three genotype groups for rs1131018. The number of individuals and cells included in each analysis can be found in the Source Data file.*

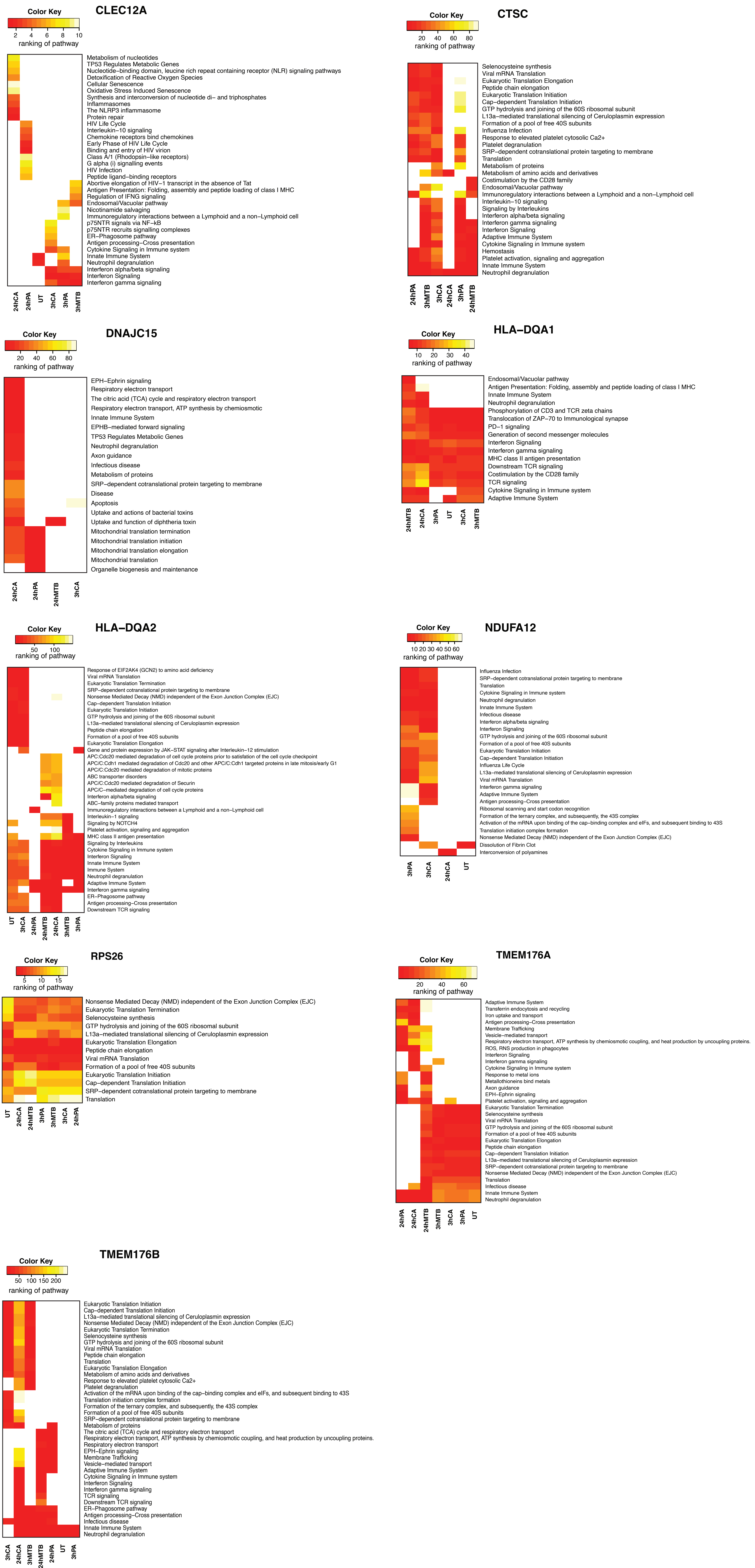

CLEC12A
Color Key
2 4 6 8 10
ranking of pathway
Metabolism of nucleotides
TP53 Regulates Metabolic Genes
Nucleotide–binding domain, leucine rich repeat containing receptor (NLR) signaling pathways
Detoxification of Reactive Oxygen Species
Cellular Senescence
Oxidative Stress Induced Senescence
Synthesis and interconversion of nucleotide di– and triphosphates
Inflammasomes
The NLRP3 inflammasome
Protein repair
HIV Life Cycle
Interleukin–10 signaling
Chemokine receptors bind chemokines
Early Phase of HIV Life Cycle
Binding and entry of HIV virion
Class A/1 (Rhodopsin–like receptors)
G alpha (i) signalling events
HIV Infection
Peptide ligand–binding receptors
Abortive elongation of HIV–1 transcript in the absence of Tat
Antigen Presentation: Folding, assembly and peptide loading of class I MHC
Endosomal/Vacuolar pathway
Nicotinamide salvaging
Immunoregulatory interactions between a Lymphoid and a non–Lymphoid cell
p75NTR signals via NF–kB
p75NTR recruits signalling complexes
ER–Phagosome pathway
Antigen processing–Cross presentation
Cytokine Signaling in Immune system
Innate Immune System
Neutrophil degranulation
Interferon alpha/beta signaling
Interferon Signaling
Interferon gamma signaling
24hCA
24hPA
UT
3hCA
3hPA
3hMTB
CTSC
Color Key
20 40 60 80
ranking of pathway
Selenocysteine synthesis
Viral mRNA Translation
Eukaryotic Translation Elongation
Peptide chain elongation
Eukaryotic Translation Initiation
Cap–dependent Translation Initiation
GTP hydrolysis and joining of the 60S ribosomal subunit
L13a–mediated translational silencing of Ceruloplasmin expression
Formation of a pool of free 40S subunits
Influenza Infection
Response to elevated platelet cytosolic Ca2+
Platelet degranulation
SRP–dependent cotranslational protein targeting to membrane
Translation
Metabolism of proteins
Metabolism of amino acids and derivatives
Costimulation by the CD28 family
Endosomal/Vacuolar pathway
Immunoregulatory interactions between a Lymphoid and a non–Lymphoid cell
Interleukin–10 signaling
Signaling by Interleukins
Interferon alpha/beta signaling
Interferon gamma signaling
Interferon Signaling
Adaptive Immune System
Cytokine Signaling in Immune system
Hemostasis
Platelet activation, signaling and aggregation
Innate Immune System
Neutrophil degranulation
24hPA
3hMTB
3hCA
24hCA
3hPA
24hMTB
DNAJC15
Color Key
20 40 60 80
ranking of pathway
EPH–Ephrin signaling
Respiratory electron transport
The citric acid (TCA) cycle and respiratory electron transport
Respiratory electron transport, ATP synthesis by chemiosmotic
Innate Immune System
EPHB–mediated forward signaling
TP53 Regulates Metabolic Genes
Neutrophil degranulation
Axon guidance
Infectious disease
Metabolism of proteins
SRP–dependent cotranslational protein targeting to membrane
Disease
Apoptosis
Uptake and actions of bacterial toxins
Uptake and function of diphtheria toxin
Mitochondrial translation termination
Mitochondrial translation initiation
Mitochondrial translation elongation
Mitochondrial translation
Organelle biogenesis and maintenance
24hCA
24hPA
24hMTB
3hCA
HLA–DQA1
Color Key
10 20 30 40
ranking of pathway
Endosomal/Vacuolar pathway
Antigen Presentation: Folding, assembly and peptide loading of class I MHC
Innate Immune System
Neutrophil degranulation
Phosphorylation of CD3 and TCR zeta chains
Translocation of ZAP–70 to Immunological synapse
PD–1 signaling
Generation of second messenger molecules
Interferon Signaling
Interferon gamma signaling
MHC class II antigen presentation
Downstream TCR signaling
Costimulation by the CD28 family
TCR signaling
Cytokine Signaling in Immune system
Adaptive Immune System
24hMTB
24hCA
3hPA
UT
3hCA
3hMTB
HLA–DQA2
Color Key
50 100
ranking of pathway
Response of EIF2AK4 (GCN2) to amino acid deficiency
Viral mRNA Translation
Eukaryotic Translation Termination
SRP–dependent cotranslational protein targeting to membrane
Nonsense Mediated Decay (NMD) independent of the Exon Junction Complex (EJC)
Cap–dependent Translation Initiation
Eukaryotic Translation Initiation
GTP hydrolysis and joining of the 60S ribosomal subunit
L13a–mediated translational silencing of Ceruloplasmin expression
Peptide chain elongation
Formation of a pool of free 40S subunits
Eukaryotic Translation Elongation
Gene and protein expression by JAK–STAT signaling after Interleukin–12 stimulation
APC:Cdc20 mediated degradation of cell cycle proteins prior to satisfation of the cell cycle checkpoint
APC/C:Cdh1 mediated degradation of Cdc20 and other APC/C:Cdh1 targeted proteins in late mitosis/early G1
APC/C:Cdc20 mediated degradation of mitotic proteins
ABC transporter disorders
APC/C:Cdc20 mediated degradation of Securin
APC/C–mediated degradation of cell cycle proteins
Interferon alpha/beta signaling
ABC–family proteins mediated transport
Immunoregulatory interactions between a Lymphoid and a non–Lymphoid cell
Interleukin–1 signaling
Signaling by NOTCH4
Platelet activation, signaling and aggregation
MHC class II antigen presentation
Signaling by Interleukins
Cytokine Signaling in Immune system
Interferon Signaling
Innate Immune System
Immune System
Neutrophil degranulation
Adaptive Immune System
Interferon gamma signaling
ER–Phagosome pathway
Antigen processing–Cross presentation
Downstream TCR signaling
UT
3hCA
24hPA
24hMTB
24hCA
3hMTB
3hPA
NDUFA12
Color Key
10 20 30 40 50 60
ranking of pathway
Influenza Infection
SRP–dependent cotranslational protein targeting to membrane
Translation
Cytokine Signaling in Immune system
Neutrophil degranulation
Innate Immune System
Infectious disease
Interferon alpha/beta signaling
Interferon Signaling
GTP hydrolysis and joining of the 60S ribosomal subunit
Formation of a pool of free 40S subunits
Eukaryotic Translation Initiation
Cap–dependent Translation Initiation
Influenza Life Cycle
L13a–mediated translational silencing of Ceruloplasmin expression
Viral mRNA Translation
Interferon gamma signaling
Adaptive Immune System
Antigen processing–Cross presentation
Ribosomal scanning and start codon recognition
Formation of the ternary complex, and subsequently, the 43S complex
Activation of the mRNA upon binding of the cap–binding complex and eIFs, and subsequent binding to 43S
Translation initiation complex formation
Nonsense Mediated Decay (NMD) independent of the Exon Junction Complex (EJC)
Dissolution of Fibrin Clot
Interconversion of polyamines
3hPA
3hCA
24hCA
UT
RPS26
Color Key
5 10 15
ranking of pathway
Nonsense Mediated Decay (NMD) independent of the Exon Junction Complex (EJC)
Eukaryotic Translation Termination
Selenocysteine synthesis
GTP hydrolysis and joining of the 60S ribosomal subunit
L13a–mediated translational silencing of Ceruloplasmin expression
Eukaryotic Translation Elongation
Peptide chain elongation
Viral mRNA Translation
Formation of a pool of free 40S subunits
Eukaryotic Translation Initiation
Cap–dependent Translation Initiation
SRP–dependent cotranslational protein targeting to membrane
Translation
UT
24hCA
24hMTB
3hPA
3hMTB
3hCA
24hPA
TMEM176A
Color Key
20 40 60
ranking of pathway
Adaptive Immune System
Transferrin endocytosis and recycling
Iron uptake and transport
Antigen processing–Cross presentation
Membrane Trafficking
Vesicle–mediated transport
Respiratory electron transport, ATP synthesis by chemiosmotic coupling, and heat production by uncoupling proteins.
ROS, RNS production in phagocytes
Interferon Signaling
Interferon gamma signaling
Cytokine Signaling in Immune system
Response to metal ions
Metallothioneins bind metals
Axon guidance
EPH–Ephrin signaling
Platelet activation, signaling and aggregation
Eukaryotic Translation Termination
Selenocysteine synthesis
Viral mRNA Translation
GTP hydrolysis and joining of the 60S ribosomal subunit
Formation of a pool of free 40S subunits
Eukaryotic Translation Elongation
Peptide chain elongation
Cap–dependent Translation Initiation
L13a–mediated translational silencing of Ceruloplasmin expression
SRP–dependent cotranslational protein targeting to membrane
Nonsense Mediated Decay (NMD) independent of the Exon Junction Complex (EJC)
Translation
Infectious disease
Innate Immune System
Neutrophil degranulation
24hPA
24hCA
24hMTB
3hMTB
3hCA
3hPA
UT
TMEM176B
Color Key
50 100 150 200
ranking of pathway
Eukaryotic Translation Initiation
Cap–dependent Translation Initiation
L13a–mediated translational silencing of Ceruloplasmin expression
Nonsense Mediated Decay (NMD) independent of the Exon Junction Complex (EJC)
Eukaryotic Translation Termination
Selenocysteine synthesis
GTP hydrolysis and joining of the 60S ribosomal subunit
Viral mRNA Translation
Peptide chain elongation
Translation
Eukaryotic Translation Elongation
Metabolism of amino acids and derivatives
Response to elevated platelet cytosolic Ca2+
Platelet degranulation
Activation of the mRNA upon binding of the cap–binding complex and eIFs, and subsequent binding to 43S
Translation initiation complex formation
Formation of the ternary complex, and subsequently, the 43S complex
Formation of a pool of free 40S subunits
SRP–dependent cotranslational protein targeting to membrane
Metabolism of proteins
The citric acid (TCA) cycle and respiratory electron transport
Respiratory electron transport, ATP synthesis by chemiosmotic coupling, and heat production by uncoupling proteins.
Respiratory electron transport
EPH–Ephrin signaling
Membrane Trafficking
Vesicle–mediated transport
Adaptive Immune System
Cytokine Signaling in Immune system
Interferon Signaling
Interferon gamma signaling
TCR signaling
Downstream TCR signaling
ER–Phagosome pathway
Antigen processing–Cross presentation
Infectious disease
Innate Immune System
Neutrophil degranulation
3hCA
24hCA
3hMTB
24hMTB
24hPA
UT
3hPA

Supplementary Figure 6. Pathway enrichment analysis of co-expression QTL gene sets

*Heatmaps showing the enrichment ranks of the pathways associated with the set of co-expressed genes affected by each co-expression QTL gene. Darker colors represent lower ranks, i.e. stronger enrichment, and lighter colors represent higher ranks, i.e. less enrichment. Full summary statistics can be found in **Supplementary Data 6**. The number of individuals and cells included in each analysis can be found in the Source Data file.*

Supplementary Note 1. Single-cell eQTLGen Consortium

Cohort collection Yoshinari Ando[1], Odmaa Bayaraa[2], Irene van Blokland[3,4], Mame M. Dieng[2], Lude Franke[4,5], M. Grace Gordon[6-9], Hilde E. Groot[3], Pim van der Harst[10], Chung-Chau Hon[1], Youssef Idaghdour[2], Francesca Luca[11,12], Vinu Manikanda[2], Jonathan Moody[1], Martijn C. Nawijn[13,14], Yukinori Okada[15,16], Woong-Yang Park[17], Roger Pique-Regi[11,12], Joseph E. Powell[18,19], Deepa Rajagopalan[20], Tala Shahin[2], Jay W. Shin[20], Gosia Trynka[21,22], Monique G.P. van der Wijst[4,5], Seyhan Yazar[18], Jimmie Ye[7,8,23-26].

Pipeline development and data analysis José Alquicira-Hernández[18,27], Christina B. Azodi[28,29], Marijn Berg[13,14], Marc Jan Bonder[30], Harm Brugge[4,5], Anna Cuomo[18,21], Ryuya Edahiro[15,31], Lude Franke[4,5], M. Grace Gordon[6-9], Matthias Heinig[32,33], Martin Hemberg[34], Niek de Klein[21,22], Maryna Korshevniuk[4,5], Jimmy Tsz Hang Lee[21], Francesca Luca[11,12], Ahmed Mahfouz[35-37], Davis J. McCarthy[28,29], Marta Melé[38], Lieke Michielsen[35-37], Drew Neavin[18], Roy Oelen[4,5], Yukinori Okada[15,16], Aida Ripoll-Cladellas[38], Roger Pique-Regi[11,12], Joseph E. Powell[18,19], Deepa Rajagopalan[20], Oliver Stegle[21,30,39], Monique G.P. van der Wijst[4,5], Chun J. Ye[7,8,23-26].

Steering team Marc Jan Bonder[30], Lude Franke[4,5], Martin Hemberg[34], Ahmed Mahfouz[35-376], Marta Melé[38], Joseph E. Powell[18,19], Monique G.P. van der Wijst[4,5].

[1] Laboratory for Genome Information Analysis, RIKEN Center for Integrative Medical Sciences, Japan.
[2] Biology Program, New York University Abu Dhabi, United Arab Emirates.
[3] Department of Cardiology, University of Groningen, University Medical Center Groningen, Groningen, the Netherlands.
[4] Department of Genetics, University of Groningen, University Medical Center Groningen.
[5] Oncode Institute, Groningen, the Netherlands.
[6] Biological and Medical Informatics Graduate Program, University of California San Francisco, San Francisco, USA.
[7] UCSF Division of Rheumatology, Department of Medicine, University of California San Francisco, San Francisco, CA, USA.
[8] Institute for Human Genetics, University of California San Francisco, USA.
[9] Department of Bioengineering and Therapeutic Sciences, University of California San Francisco, San Francisco, USA.
[10] Department of Cardiology, University Medical Center Utrecht, Utrecht, the Netherlands.
[11] Center for Molecular Medicine and Genetics, Wayne State University School of Medicine, Detroit, USA.
[12] Department of Obstetrics and Gynecology, Wayne State University School of Medicine, Detroit, USA.
[13] Department of Pathology and Medical Biology, University of Groningen, University Medical Center Groningen, Groningen, the Netherlands.
[14] GRIAC research institute, University Medical Center Groningen, Groningen, the Netherlands.
[15] Department of Statistical Genetics, Osaka University Graduate School of Medicine.
[16] Laboratory for Systems Genetics, RIKEN Center for Integrative Medical Sciences.
[17] Samsung Genome Institute, Samsung Medical Center, Seoul, Korea.

[18] Garvan-Weizmann Centre for Cellular Genomics, Garvan Institute of Medical Research, Sydney, Australia.
[19] UNSW Cellular Genomics Futures Institute, University of New South Wales, Sydney, Australia.
[20] A*STAR Genome Institute of Singapore, Singapore.
[21] Wellcome Sanger Institute, Wellcome Genome Campus, Cambridge, UK.
[22] Open Targets, Wellcome Genome Campus, Cambridge, UK.
[23] Bakar Computational Health Sciences Institute, University of California San Francisco, San Francisco, CA, USA.
[24] Department of Epidemiology and Biostatistics, University of California San Francisco, San Francisco, CA, USA.
[25] Parker Institute for Cancer Immunotherapy, San Francisco, CA, USA.
[26] Chan Zuckerberg Biohub, San Francisco, CA, USA
[27] Computational Genomics, Institute for Molecular Bioscience, University of Queensland, Brisbane, Australia
[28] St. Vincent's Institute of Medical Research, Fitzroy, Victoria, Australia
[29] University of Melbourne, Parkville, Victoria, Australia
[30] Division of Computational Genomics and Systems Genetics, German Cancer Research Center (DKFZ), Heidelberg, Germany.
[31] Department of Respiratory Medicine and Clinical Immunology, Osaka University Graduate School of Medicine.
[32] Computational Health Center, Helmholtz Zentrum München, Deutsches Forschungszentrum für Gesundheit und Umwelt (GmbH), Munich, Germany.
[33] Department of Informatics, Technical University of Munich, Munich, Germany.
[34] Evergrande Center for Immunologic Disease, Harvard Medical School and Brigham and Women's Hospital, Boston, USA.
[35] Department of Human Genetics, Leiden University Medical Center, Leiden, The Netherlands.
[36] Leiden Computational Biology Center, Leiden University Medical Center, Leiden, The Netherlands.
[37] Delft Bioinformatics Laboratory, Delft University of Technology, Delft, The Netherlands
[38] Life Sciences Department, Barcelona Supercomputing Center, Barcelona, Catalonia, Spain
[39] European Molecular Biology Laboratory (EMBL), Genome Biology Unit, Heidelberg, Germany.

Supplementary Note 2. BIOS Consortium
(Biobank-based Integrative Omics Study)

Management Team Bastiaan T. Heijmans (chair)[1], Peter A.C. ’t Hoen[2], Joyce van Meurs[3], Aaron Isaacs[4], Rick Jansen[5], Lude Franke[6].

Cohort collection Dorret I. Boomsma[7], René Pool[7], Jenny van Dongen[7], Jouke J. Hottenga[7] (Netherlands Twin Register); Marleen MJ van Greevenbroek[8], Coen D.A. Stehouwer[8], Carla J.H. van der Kallen[8], Casper G. Schalkwijk[8] (Cohort study on Diabetes and Atherosclerosis Maastricht); Cisca Wijmenga[6], Lude Franke[6], Sasha Zhernakova[6], Ettje F. Tigchelaar[6] (LifeLines Deep); P. Eline Slagboom[1], Marian Beekman[1], Joris Deelen[1], Diana van Heemst[9] (Leiden Longevity Study); Jan H. Veldink[10], Leonard H. van den Berg[10] (Prospective ALS Study Netherlands); Cornelia M. van Duijn[4], Bert A. Hofman[11], Aaron Isaacs[4], André G. Uitterlinden[3] (Rotterdam Study).

Data Generation Joyce van Meurs (Chair)[3], P. Mila Jhamai[3], Michael Verbiest[3], H. Eka D. Suchiman[1], Marijn Verkerk[3], Ruud van der Breggen[1], Jeroen van Rooij[3], Nico Lakenberg[1].

Data management and computational infrastructure Hailiang Mei (Chair)[12], Maarten van Iterson[1], Michiel van Galen[2], Jan Bot[13], Dasha V. Zhernakova[6], Rick Jansen[5], Peter van ’t Hof[12], Patrick Deelen[6], Irene Nooren[13], Peter A.C. ’t Hoen[2], Bastiaan T. Heijmans[1], Matthijs Moed[1].

Data Analysis Lude Franke (Co-Chair)[6], Martijn Vermaat[2], Dasha V. Zhernakova[6], René Luijk[1], Marc Jan Bonder[6], Maarten van Iterson[1], Patrick Deelen[6], Freerk van Dijk[14], Michiel van Galen[2], Wibowo Arindrarto[12], Szymon M. Kielbasa[15], Morris A. Swertz[14], Erik. W van Zwet[15], Rick Jansen[5], Peter-Bram ’t Hoen (Co-Chair)[2], Bastiaan T. Heijmans (Co-Chair)[1].

[1] Molecular Epidemiology Section, Department of Medical Statistics and Bioinformatics, Leiden University Medical Center, Leiden, The Netherlands
[2] Department of Human Genetics, Leiden University Medical Center, Leiden, The Netherlands
[3] Department of Internal Medicine, ErasmusMC, Rotterdam, The Netherlands
[4] Department of Genetic Epidemiology, ErasmusMC, Rotterdam, The Netherlands
[5] Department of Psychiatry, VU University Medical Center, Neuroscience Campus Amsterdam, Amsterdam, The Netherlands
[6] Department of Genetics, University of Groningen, University Medical Centre Groningen, Groningen, The Netherlands
[7] Department of Biological Psychology, VU University Amsterdam, Neuroscience Campus Amsterdam, Amsterdam, The Netherlands
[8] Department of Internal Medicine and School for Cardiovascular Diseases (CARIM), Maastricht University Medical Center, Maastricht, The Netherlands
[9] Department of Gerontology and Geriatrics, Leiden University Medical Center, Leiden, The Netherlands
[10] Department of Neurology, Brain Center Rudolf Magnus, University Medical Center Utrecht, Utrecht, The Netherlands
[11] Department of Epidemiology, ErasmusMC, Rotterdam, The Netherlands

[12] Sequence Analysis Support Core, Leiden University Medical Center, Leiden, The Netherlands
[13] SURFsara, Amsterdam, the Netherlands
[14] Genomics Coordination Center, University Medical Center Groningen, University of Groningen, Groningen, the Netherlands
[15] Medical Statistics Section, Department of Medical Statistics and Bioinformatics, Leiden University Medical Center, Leiden, The Netherlands
