# Supplementary material for: Single-cell RNA-sequencing of peripheral blood mononuclear cells reveals widespread, context-specific gene expression regulation upon pathogenic exposure
Source: Nat Commun. 2022 Jun 7;13:3267. doi: 10.1038/s41467-022-30893-5 (PMC9174272; doi:10.1038/s41467-022-30893-5)
Supplement: Supplementary file 3 — Description of Additional Supplementary Files [file 41467_2022_30893_MOESM3_ESM.pdf]

Description of Additional Supplementary Files

Supplementary Data 1. Sample metadata

The first tab ('1M-scBloodNL_metadata') provides phenotypical metadata of the 120 individuals included in this study. Tab 2 ('labels') provides an explanation for the column names in tab 1. The third tab ('DutchPopulation') shows the prevalence of a list of immune-mediated diseases in the Dutch population.

Supplementary Data 2. Cellranger metadata

Output of the Cellranger pipeline per scRNA-seq library sample (pool of 8 individuals). Samples colored red were removed after quality control.

Supplementary Data 3. Quality control

The sequential steps by which the cells are quality control filtered are shown and the number of cells lost during these steps are indicated.

Supplementary Data 4. Cell type markers

Marker genes used for cell type annotation of clusters.

Supplementary Data 5. Differential Gene expression summary statistics

Differential expression results in the low- and high-resolution cell types. Per tab one cell type is shown. The Bonferroni-corrected meta-analysis fold change and p-values of all genes that are significantly differential expressed (Bonferroni-corrected version-chemistry meta-analysis p-value < 0.05) in at least one stimulation-timepoint combination of that cell type are listed.

Supplementary Data 6. Pathway analysis summary statistics

Significant pathway analysis (BH-corrected p < 0.05) results per low- and high-resolution cell type using the corresponding upregulated genes as shown in Table S5.

Supplementary Data 7. eQTL mapping summary statistics eQTLgen lead-eSNP eQTL

All cis-eQTL results (Z-score indicating strength and direction of the effect for given alternate allele) that were significant (FDR-corrected p < 0.05, indicated with an asterisk) in at least

one timepoint-stimulation combination across all major and subcell types (each tab represents one major cell type that is also analyzed separately by subtype, or a bulk-like sample combining all cells per individual). The nominal p-value of the bulk-like untreated (UT) condition is shown in each tab as a reference to compare cell-type-specific results against. The eQTL analysis was confined to those SNPs (MAF > 0.1) that were significant lead-eSNPs in bulk whole blood eQTLGen meta-analysis[37].

Supplementary Data 8. eQTL mapping summary statistics Genome-wide

The cis-eQTL results (Z-score indicating strength and direction of the effect for given alternate allele) of all eQTL genes that were significant (FDR-corrected $p < 0.05$, indicated with an asterisk) in at least one timepoint-stimulation combination across all cell types (each tab represents one cell type, or a bulk-like sample combining all cells per individual). All SNPs (MAF > 0.1) were tested in this analysis, the lead-eSNP per timepoint-stimulation combination is provided.

Supplementary Data 9. Response-QTL mapping analysis summary statistics

The response-QTL results (Z-score indicating strength and direction of the effect for given alternate allele) that were significant (FDR-corrected $p < 0.05$, indicated with an asterisk) in at least one timepoint-stimulation combination across all cell types (each tab represents one cell type, or a bulk-like sample combining all cells per individual). The nominal p-value of the bulk-like untreated (UT) condition is shown in each tab as a reference to compare cell-type-specific results against. The response-QTL analysis was confined to those SNPs that were significant lead-eSNPs in at least one stimulation-timepoint combination and cell type in the eQTLGen[37]-confined analysis (from table S7).

Supplementary Data 10. Genomic inflation analysis

The amount of genomic inflation (lambda) of eQTL, non-response QTL and response-QTL SNPs based on six immune-mediated disease GWASes (separated per tab). The number of tested SNPs is reported for each category (eQTL, non-response QTL and response-QTL).

Supplementary Data 11. Co-expression QTL analysis in monocytes summary statistics

The co-expression QTL results in the monocytes from the 1M-scBloodNL dataset that are significant (10x Genomics version chemistry meta-analysis p < permutation-based significance threshold (2nd row of each tab)) in at least one stimulation-timepoint combination are shown. Spearman r correlations are shown for the significant co-expression QTLs, separated by 10x Genomics version chemistry.

Supplementary Data 12. Co-expression QTL analysis SLE cohort in monocytes summary statistics

The gene set that was significantly co-expressed with rs12230244-CLEC12A in the monocytes from the 1M-scBloodNL cohort (Table S11) was tested in an independent SLE and healthy control cohort. A permutation-based significance threshold (2nd row) was used to determine whether the meta-analysis p-value across all SLE or all healthy control sub-cohorts was significant. The spearman r correlation in each of the individual cohorts is indicated. The last column shows whether the co-expressed gene is part of the REACTOME interferon signaling pathway.
