# Supplementary material for: Single-cell RNA-sequencing of peripheral blood mononuclear cells reveals widespread, context-specific gene expression regulation upon pathogenic exposure
Source: Nat Commun. 2022 Jun 7;13:3267. doi: 10.1038/s41467-022-30893-5 (PMC9174272; doi:10.1038/s41467-022-30893-5)
Supplement: Supplementary file 16 — Reporting Summary [file 41467_2022_30893_MOESM16_ESM.pdf]

nature portfolio

Corresponding author(s): Monique G.P. van der Wijst, Lude Franke

Last updated by author(s): Feb 18, 2022

Reporting Summary

Nature Portfolio wishes to improve the reproducibility of the work that we publish. This form provides structure for consistency and transparency in reporting. For further information on Nature Portfolio policies, see our Editorial Policies and the Editorial Policy Checklist.

Statistics

For all statistical analyses, confirm that the following items are present in the figure legend, table legend, main text, or Methods section.

n/a	Confirmed	
☐	☒	The exact sample size (n) for each experimental group/condition, given as a discrete number and unit of measurement
☐	☒	A statement on whether measurements were taken from distinct samples or whether the same sample was measured repeatedly
☐	☒	The statistical test(s) used AND whether they are one- or two-sided<br>*Only common tests should be described solely by name; describe more complex techniques in the Methods section.*
☐	☒	A description of all covariates tested
☐	☒	A description of any assumptions or corrections, such as tests of normality and adjustment for multiple comparisons
☐	☒	A full description of the statistical parameters including central tendency (e.g. means) or other basic estimates (e.g. regression coefficient) AND variation (e.g. standard deviation) or associated estimates of uncertainty (e.g. confidence intervals)
☐	☒	For null hypothesis testing, the test statistic (e.g. F, t, r) with confidence intervals, effect sizes, degrees of freedom and P value noted<br>*Give P values as exact values whenever suitable.*
☒	☐	For Bayesian analysis, information on the choice of priors and Markov chain Monte Carlo settings
☒	☐	For hierarchical and complex designs, identification of the appropriate level for tests and full reporting of outcomes
☐	☒	Estimates of effect sizes (e.g. Cohen's d, Pearson's r), indicating how they were calculated

Our web collection on statistics for biologists contains articles on many of the points above.

Software and code

Policy information about availability of computer code

Data collection	No software was used for data collection.
Data analysis	The original code for CellRanger v3.0.2 (https://support.10xgenomics.com/single-cell-gene-expression/software/pipelines/latest/what-is-cell-ranger), Seurat v3.1 (https://github.com/satijalab/seurat), Eagle v2.322 (https://github.com/poruloh/Eagle), Demuxlet 85dca0a4d648d18e6b240a2298672394fe10c6e6 (Mar 25 2019) (https://github.com/statgen/demuxlet), Souporcell v1.0 (https://github.com/wheaton5/souporcell), Freemuxlet v1 as part of the Popscle suite of statistical genetics tools (https://github.com/statgen/popscle), Scrublet v0.2 (https://github.com/swolock/scrublet), and our in-house eQTL pipeline v1.4.0 (https://github.com/molgenis/systemsgenetics/tree/master/eqtl-mapping-pipeline) can be found at GitHub. All custom-written code is made available via GitHub (https://github.com/molgenis/1M-cells).

For manuscripts utilizing custom algorithms or software that are central to the research but not yet described in published literature, software must be made available to editors and reviewers. We strongly encourage code deposition in a community repository (e.g. GitHub). See the Nature Portfolio guidelines for submitting code & software for further information.

Data

Policy information about availability of data

All manuscripts must include a data availability statement. This statement should provide the following information, where applicable:

- Accession codes, unique identifiers, or web links for publicly available datasets
- A description of any restrictions on data availability
- For clinical datasets or third party data, please ensure that the statement adheres to our policy

Raw gene expression counts, eQTL and co-expression QTL summary statistics can be found under “Supplementary Data” at the website accompanying this paper

(https://eqtlgen.org/sc/datasets/1m-scbloodnl.html). Processed (de-anonymized) scRNA-seq data, including a text file that links each cell barcode to its respective individual, has been deposited at the European Genome-Phenome Archive (EGA), which is hosted by the EBI and the CRG, under accession number EGAS00001005376 Gene expression and genotype data can be obtained and requested by filling in a short web form at https://eqtlgen.org/sc/datasets/1m-scbloodnl.html. This form is subsequently reviewed by a single Data Access Committee, who will be able to approve access to both the raw gene expression and genotype data within 5 working days (during the holiday season there might be a slight delay). Once the proposed research is approved, access to the relevant gene expression or genotyped data will be free of charge. Access to the genotype and gene expression data is facilitated via the HPC cluster of the UMCG and the EGA, respectively. Access to this data is restricted to comply with the European Union General Data Protection Regulation for protection of privacy-sensitive data. Sample metadata (age, gender) is presented in Table S1. The REACTOME and TRANSFAC release 2020.2 v253 database can be accessed through https://reactome.org/ and https://biit.cs.ut.ee/gprofiler/gost, respectively.

Field-specific reporting

Please select the one below that is the best fit for your research. If you are not sure, read the appropriate sections before making your selection.

☒ Life sciences ☐ Behavioural & social sciences ☐ Ecological, evolutionary & environmental sciences

For a reference copy of the document with all sections, see nature.com/documents/nr-reporting-summary-flat.pdf

Life sciences study design

All studies must disclose on these points even when the disclosure is negative.

Sample size

1.3M single-cells coming from 120 donors were included in this study. Based on previous experience (van der Wijst et al. 2018, Nature Genetics), we know that 25,000 cells coming from 45 donors are sufficient, but at the lower end of what is needed, to conduct an eQTL and co-expression QTL analysis in single-cell data.

Data exclusions

Cells with less than 200 detected genes were discarded, as were other low-quality cells (i.e. clusters of cells with a low number of expressed genes and a relatively high mitochondrial content, or missed, likely same-individual, doublets). As a consequence of QC dropouts, the number of included individuals varied among the stimulation–timepoint combinations (UT: 104 individuals, 3h CA: 120 individuals, 3h MTB: 104 individuals, 3h PA: 112 individuals, 24h CA: 119 individuals, 24h MTB: 112 individuals, 24h PA: 111 individuals).

For the SLE scRNA-seq data: monocytes with less than 1500 UMIs were removed, as were donors with fewer than 200 cells remaining after applying this cutoff. These cut-offs were chosen to ensure robust co-expression QTL analysis could be performed and that better resembles our own dataset.

Replication

Source data contains information on the number of individuals and cells that were included in the study (separated per cell type and timepoint-stimulation condition).
DE analysis: concordance with 24h CA stimulation DE results from de Vries et al. 2020 - Plos Pathogens.
eQTL analysis: concordance check with bulk whole blood eQLTGen data from Võsa et al. 2018 - biorXiv
co-expression QTL analysis: concordance check with CD4+T RPS26 co-expression QTLs from van der Wijst et al. 2018 - Nature Genetics, replication of IFN-modulated CLEC12A co-expression QTL effects with SLE PRS scores from BIOS whole blood data and SLE patient scRNA-seq cohort (GEO accession number: GSE174188).

Randomization

For each donor we have generated the same set of unstimulated and pathogen-stimulated conditions. 8 donors were pooled in 1 sample batch, trying to balance both sexes, otherwise this pool of 8 donors was generated randomly.

Blinding

Blinding was not necessary during data collection, because we worked with sample pools of 8 donors that in general contained 2 different stimulation-timepoint combinations.

Reporting for specific materials, systems and methods

We require information from authors about some types of materials, experimental systems and methods used in many studies. Here, indicate whether each material, system or method listed is relevant to your study. If you are not sure if a list item applies to your research, read the appropriate section before selecting a response.

Materials & experimental systems

n/a	Involved in the study
☒	☐ Antibodies
☒	☐ Eukaryotic cell lines
☒	☐ Palaeontology and archaeology
☒	☐ Animals and other organisms
☐	☒ Human research participants
☒	☐ Clinical data
☒	☐ Dual use research of concern

Methods

n/a	Involved in the study
☒	☐ ChIP-seq
☒	☐ Flow cytometry
☒	☐ MRI-based neuroimaging

Human research participants

Policy information about studies involving human research participants

Population characteristics	120 individuals from the Northern Netherlands population cohort Lifelines. Age: 20-79 years. Gender: 67F, 53M. See Table S1 for exact details.
Recruitment	The 120 individuals were recruited through invitation by mail. Only those individuals that were also included in the Lifelines DEEP cohort (Tichelaar et al. 2015 - BMJ Open), lived (according to documentation) within a range of 50km from the UMCG in Groningen, and did not participate in our previous study (van der Wijst et al. 2018 - Nature Genetics), were invited. As we required individuals to visit the hospital (UMCG) for collection of their blood and joining the study was voluntary, we may have introduced selection bias (mobile individuals, positive attitude towards science).
Ethics oversight	The LifeLines DEEP study was approved by the ethics committee of the University Medical Centre Groningen, document number METC UMCG LLDEEP: M12.113965. All participants signed an informed consent form prior to study enrollment. All procedures performed in studies involving human participants were in accordance with the ethical standards of the institutional and/or national research committee and with the 1964 Helsinki declaration and its later amendments or comparable ethical standards.

Note that full information on the approval of the study protocol must also be provided in the manuscript.
